# Practice of Medicine

**Published:** 1858-09

**Authors:** 


### PRACTICE OF MEDICINE.

Observations on the Use of the Shower-bath in Delirium Tremens, and other similar Cases of Cerebral Excitement. By *Robert Law, M.D.*, Professor of the Institutes of Medicine in the School of Physic in Ireland, etc.—The following observations were made when delirium tremens was a very common disease among us. Subsequently the "temperance movement" produced such a change in the habits of our countrymen, among whom this disease chiefly prevailed, that an instance of it seldom presented itself to the physician. I felt that the time for the recommendation of any therapeutic agent applicable to the disease had gone by. But, unhappily, the effects of the temperance movement were but temporary. Intoxicating liquors are now as freely used as they ever were, and delirium tremens is now as common as it ever was, and a remedy of any value in it is now as well deserving of being made known as it ever was.

It will be readily admitted that in no case, and under no circumstances, does cerebral excitement assume so formidable an aspect as it does not unfrequently in delirium tremens, and there is no case where high excitement is oftener attended with speedily fatal collapse. I shall not soon forget a case which supplied me with an illustration of this fact. I admitted into the hospital a young man whose friends, that accompanied him, declared to be affected with delirium tremens. He answered questions most coherently, and in such a way that I should have doubted the truth of their statement respecting him, were I not well aware how often the disease is suspended during the day to break out with violence at night. This proved to be the case in the present instance: the person so tranquil during the day became so outrageous at night that he was obliged to be placed in a separate apartment, and required the porter to remain with him to keep him quiet. About midnight he made a strong effort to get out of bed, but after a long struggle the porter succeeded in mastering him, and left him, as he supposed, disposed to sleep. He returned in two hours, and found him quite dead. Nor is this a solitary instance, in my experience, of sudden sinking very speedily following upon the excitement of delirium tremens. The delirium caused by the excessive or continued use of ardent spirits has long since proved itself refractory to the treatment ordinarily and successfully employed against it when occurring under other circumstances, and in other diseases. Although opium may be considered to be its general remedy, yet many are the cases in which it not only fails to be useful, but, I am persuaded, has been productive of positive mischief. Tartar emetic, either alone or combined with opium in varying proportions, although it, too, has often been successfully employed against it, yet, in many instances, has disappointed my expectations. It was the failure of these means, and the feeling of the necessity of adopting some means calculated to produce an immediate impression upon a disease that did not admit of delay, that suggested to me the shower-bath. Nor was it alone in cases of pure delirium tremens that I used it, but also in the delirium of fever, in persons addicted to the use of ardent spirits. I shall now detail some cases to exhibit the effects of it.

Case I.—Frederick Bridge, aged thirty-two, writing-clerk, admitted into hospital January 31st. For a week previous to his coming into hospital he exhibited a peculiar fidgety, restless manner, and, three days ago, when getting out of bed, he fell down in a fit, attended with convulsions, from which he soon recovered, and into which he relapsed three times at very short intervals. He is reported not to have slept for several nights. He is said not to be a determined drunkard, but has seldom confined himself within the strict limits of sobriety. He was reported to have passed the night previous to his admission, running through the house, hunting animals, the creatures of his disordered imagination. Phenomena on admission:—Pulse 126, feeble; skin cold and clammy; a restlessness of manner. He has no headache, nor suffusion of eyes, nor flush of face. Tongue clean and tremulous. He has a general tremor of the body. He was ordered to take a tablespoonful of the following mixture every hour until sleep resulted:—Camphor mixture, five ounces; muriate of morphia, two grains; spirit of nitrous ether, two drachms; and syrup of lemons, six drachms; and to have a tumbler of punch, his favorite beverage.

February 1st. He was very much excited during the night, and scarcely remained an instant in bed, pulling and tossing everything about. Pulse fuller and stronger; head hot; he has taken one bottle of the mixture, and half of another, without exhibiting the least appearance of being under its influence. To have six grains of tartarized antimony added to the mixture. Let him have a cold shower-bath immediately.

2d. 11 o'clock A.M. He is in a most tranquil sleep, without any stertor. He got the shower-bath at 4 o'clock yesterday, and almost immediately afterwards fell into the sleep in which he now is. 1 o'clock P.M. He has now just awoke; his mind is quite collected; he has no headache, no tremor of the body; pulse 90, soft and regular. He has, in a very remarkable degree, the peculiar fusty smell which we so often perceive in such cases. In order to make assurance sure, I directed his medicine to be repeated.

3d. He continued perfectly composed, without even any confusion of mind, and has had a quiet, tranquil night.

4th. Convalescent.

CASE II.—James Connor, aged forty-two, a habitual drinker of ardent spirits and punch, but seldom to intoxication, was admitted into hospital, late yesterday evening, April 27. He raved incessantly all night about his occupation of clothes-vender. Present phenomena:—Pulse 114; skin moist and clammy; eyes suffused; tongue covered with whitish slimy mucus; head hot; bowels torpid. He has had leeches applied to his temples before admission. He was ordered to take an ounce of the following mixture every third hour:—Camphor mixture, seven and a half ounces; tartar emetic, four grains; vinegar of opium, twenty-five drops; and spirit of nitrous ether, half an ounce. A cold shower-bath immediately. Should his condition be not improved toward evening, let six leeches be applied behind each ear, cold wash to the forehead, and his legs be stuped.

28th. He has had a most quiet, undisturbed night, without any delirium. Immediately after he got the shower-bath he fell asleep, and slept for three hours. When he awoke he requested to have the shower-bath repeated, and, after getting it, he again fell asleep, and did not awake through the night. He got the medi cine but twice, nor was anything else done for him. Pulse 120; tongue white and slimy. I neglected to mention that he was ordered a turpentine enema, which freely affected his bowels. To take an ounce every third hour of the following mixture:—Camphor mixture, seven and a half ounces; spirit of nitrous ether, half an ounce.

29th. He slept well through the night; pulse less frequent; tongue cleaner.

May 1st. He perspired freely through the night; tongue quite clean. Convalescent.

CASE III.—An elderly man, Reilly, had been under my care in hospital for some time, laboring under an obstinate cutaneous affection, when he exhibited symptoms of fever which prevailed in an epidemic form at the time. When I came to see him in the morning, the nurse told me that he had not slept an instant during the night; that his tongue had been going incessantly, uttering the most incoherent nonsense. I found him in the same loquacious mood. His face was flushed, and eyes suffused; skin hot and dusky; pulse very rapid and small; tongue red and dry. For a short time previous to the exhibition of these symptoms he had been affected with diarrhœa, which had yielded to medicine. Reilly's habits not having been the most temperate, coupled with the character of his delirium, led me to regard his case as one of the prevailing epidemic typhoid fever, modified by delirium tremens. I accordingly directed for him the following medicine:—Camphor mixture, seven and a half ounces; spirit of nitrous ether, three drachms; vinegar of opium, thirty drops; tartar emetic, one grain; mix: two tablespoonsful to be taken every third hour. Shower-bath immediately. As I apprehended that he would be chilled by the bath, I directed for him a hot drink immediately afterwards and a jar of warm water to be applied to his feet. He was seized with a slight rigor, but which soon ceased. In a very short time after he had the bath, and had taken one dose of the medicine, he fell asleep, and awoke with his mind quite composed, after having had four hours of refreshing sleep. He now went regularly through a tedious fever, but had no return of delirium. His exhaustion was extreme, and he required to be supported by stimulants, wine, ammonia, etc.

CASE IV.—James Nugent, aged thirty-four, locksmith, admitted into hospital June 13, exhibiting the following symptoms:—Pulse 130, full and strong; tongue clean; a nervous tremor of the entire body; profuse general perspiration; eyes not suffused. He had been but a very short time in the hospital when he became so violent as to require to have the strait-waistcoat immediately put on. The account that the friends who accompanied him to the hospital gave of him was, that he had been drinking for several days, and afterwards, while at breakfast, he was suddenly seized with a kind of fit, and lost all consciousness; his stomach then became sick; he had a return of the fit, and then became delirious. He was ordered to take every third hour an ounce of the following mixture:—Camphor mixture, five and a half ounces; tartar emetic, six grains; spirit of nitrous ether, three drachms; tincture of opium, thirty drops; mix. Cold shower-bath immediately, and let it be repeated in the evening, if necessary.

14th. Pulse 78, soft. He is perfectly tranquil and collected at present, and quite conscious of his position. He felt immediately refreshed by the shower-bath. His skin is cool; eyes slightly injected. The medicine caused no sickness. The mixture to be repeated; the tincture of opium to be diminished to twenty drops.

15th. Pulse 78. He is quite collected, and slept well during the night.

16th. Is quite well.

This individual came under my care again under similar circumstances, and so conscious was he of the benefit he before derived from the shower-bath, that he begged he might have it again. His request was complied with, and with as beneficial effects as before. He became perfectly quiet, and slept.

CASE V.—John Maguire, aged thirty-five, of intemperate habits, was brought to the hospital in a state of the highest excitement. The account given of him by his friends was, that he had been laboring under a violent pain in the calf of his left leg, which seized him suddenly, without his having received any external injury; that he had applied to a surgeon, who directed for him a liniment which he rubbed to his leg; that the pain left his leg, but he was soon after seized with pain in the head, and then became violently excited. Of this excitement there had been no abatement from the time of its commencement until he was admitted into hospital. We were obliged to put the strait-waistcoat on him immediately to control him, as otherwise it would have been utterly impossible to have kept him in bed. His appearance exhibited great wildness, nor would he give us the least information as to whether he felt any pain or uneasiness. I directed a cold shower-bath for him at once, and ordered him to have the following mixture:—Camphor mixture, seven ounces; tartar emetic, four grains; sedative liquor of opium, thirty drops; syrup of saffron, one ounce; mix: one ounce to be taken every third hour. After he got the shower-bath the strait-waistcoat was not again put on him, nor was there any necessity for it; he became perfectly tranquil, and confessed himself relieved from a most distressing headache, which he now admitted that he had. After about four hours he felt the pain of his head returning, when he requested that he might again have the shower-bath. It was repeated, and shortly afterwards he fell asleep, and enjoyed a night of uninterrupted rest. He had no return either of headache or delirium; and in a few days he was quite well.

I shall content myself with the details of one more case, which strikingly proved the tranquilizing effect of the bath when the patient was under the highest degree of excitement.

CASE VI.—Matthew Jones, aged forty-five, waiter in a hotel; of intemperate habits, although he was reported not to have exceeded lately; for a week previous to admission, was under treatment for an affection of his chest. I had no opportunity of seeing him until the day after his admission into hospital, when I found him in such an excited state as that with difficulty could he be kept in bed. He had been constantly getting up in quest of whisky. He had no sleep during the night. His eyes were suffused; expression peculiarly wild; pulse 108, full and strong. He talks incessantly about his occupation as a waiter. I ordered him a shower-bath immediately, and afterwards to have the following mixture:—Muriate of morphia, two grains; camphor mixture, seven ounces; spirit of nitrous ether, three drachms; syrup of saffron, five drachms; mix: two tablespoonsful to be taken every third hour. He offered the most violent resistance to the shower-bath; however, the porter, after having received some hard blows from him, succeeded in giving it to him. He became perfectly quiet after it, and remained so for three hours, when he again became excited; the shower-bath was repeated, when he again became quiet and fell asleep, and awoke in the morning perfectly sensible. He had no return of delirium through his illness, and recovered so much that I indulged a hope of his final recovery; however, I soon perceived the fetor, both from his breath and from the sputa, that announced gangrene of the lung, and discovered a large cavity, indicated by pectoriloquy, gargouillement, and cavernous respiration under the right clavicle. He soon began to sink, and died about ten days from the time of his admission into hospital. Examination of the body exhibited a large cavity, whose internal surface was irregular and sloughy, and which occupied the entire of the upper lobe of the right lung. The left lung was quite healthy.

I deem it superfluous to adduce more cases to prove the efficacy of the shower-bath in allaying cerebral excitement, and procuring sleep in delirium tremens. Nor is my experience of its value confined to pure delirium tremens; I have found it no less efficacious in the delirium of fever, when the subject of it has been of intemperate habits, and in whom the fever has been modified by the delirium tremens. In some of these cases, where there has been much prostration of strength, and the pulse very feeble, I have deemed it a measure of prudence to have at hand either a stimulating draught, composed of camphor mixture, ether, and aromatic spirit of ammonia, or hot punch, or mulled wine, to give to the patient immediately after his getting the bath, to prevent an injurious chill, and to promote re-action. I also have directed a jar of warm water to be applied to the feet, and the legs to be wrapped in hot flannels. With these precautions I have not seen a single instance of injury resulting from the bath, and have proved its most beneficial effects even in cases in which the fever has been of a low typhoid type, with the surface covered with petechiæ. Nor have I been deterred from using the bath when an affection of the chest has been present; I have only in such cases thought it prudent to have the chill taken off the water. In many cases I have found it necessary to repeat the bath from the excitement only being suspended by its single administration; and even in some cases, when the excitement has not returned, but has left behind it a wakefulness, I have found its repetition procure sleep. The patient generally experiences great relief from it. Maguire felt that the violent pain of his head at once left him after it. When the shower-bath could not be so readily procured, or where some circumstance connected with the patient hindered his getting it, I have derived considerable advantage from pouring a large quantity of cold water on his head, held over a vessel placed beside the bed. I am not aware of the shower-bath having been employed under the circumstances that I have here recommended it. And I do venture to recommend it with the utmost confidence. Nor need its use interfere with any other means that we may wish to employ. We can, at the same time, exhibit either opium simply, or combined with tartar emetic, as the circumstances of the case may seem to require. I would attribute the advantage of the bath in these cases to its threefold effect, viz.: first, the shock; second, its diverting from the head; and third, determining to the surface generally. I impute no small share of the benefit to the shock; it produces a sudden and strong impression, and seems, as it were, to cause a revolution in the train of morbid action; while the sedative effect of the cold applied to the head, relieves it, and from a partial produces a general determination to the surface, and thus restores the balance of the circulation.—*Dublin Quarterly Journ. of Med. Science*, Feb. 1858.

